# 5-Hydroxymethylcytosine in circulating cell-free DNA as a potential diagnostic biomarker for SLE

**DOI:** 10.1136/lupus-2024-001286

**Published:** 2024-10-04

**Authors:** Xinya Tong, Wenwen Chen, Lele Ye, Yanling Xiong, Yuan Xu, Yunhui Luo, Xinhang Xia, Zexia Xu, Yutong Lin, Xinqi Zhu, Nan Wang, Xiangyang Xue, Huidi Zhang, Gangqiang Guo

**Affiliations:** 1 Wenzhou Collaborative Innovation Center of Gastrointestinal Cancer in Basic Research and Precision Medicine, Wenzhou Key Laboratory of Cancer-related Pathogens and Immunity, Department of Microbiology and Immunology, Institute of Molecular Virology and Immunology, Institute of Tropical Medicine, School of Basic Medical Sciences, Wenzhou Medical University, Wenzhou, Zhejiang, China; 2 Department of Gynecology, The First Affiliated Hospital, Wenzhou Medical University, Wenzhou, Zhejiang, China; 3 Department of Nephrology, The First Affiliated Hospital, Wenzhou Medical University, Wenzhou, Zhejiang, China; 4 First Clinical College, Wenzhou Medical University, Wenzhou, Zhejiang, China

**Keywords:** Autoimmune Diseases, Lupus Erythematosus, Systemic, Autoimmunity

## Abstract

**Background:**

SLE is a complex autoimmune disease with heterogeneous manifestations and unpredictable outcomes. Early diagnosis is challenging due to non-specific symptoms, and current treatments only manage symptoms. Epigenetic alternations, including 5-Hydroxymethylome (5hmC) modifications, are important contributors to SLE pathogenesis. However, the 5hmC modification status in circulating cell-free DNA (cfDNA) of patients with SLE remains largely unexplored. We investigated the distribution of 5hmC in cfDNA of patients with SLE and healthy controls (HCs), and explored its potential as an SLE diagnosis marker.

**Methods:**

We used 5hmC-Seal to generate genome-wide 5hmC profiles of plasma cfDNA and bioinformatics analysis to screen differentially hydroxymethylated regions (DhMRs). In vitro mechanistic exploration was conducted to investigate the regulatory effect of CCCTC-binding factor (CTCF) in 5hmC candidate biomarkers.

**Results:**

We found distinct differences in genomic regions and 5hmC modification motif patterns between patients with SLE and HCs, varying with disease progression. Increased 5hmC modification enrichment was detected in SLE. Additionally, we screened 151 genes with hyper-5hmC, which are significantly involved in SLE-related processes, and 5hmC-modified *BCL2*, *CD83*, *ETS1* and *GZMB* as SLE biomarkers. Our findings suggest that CTCF regulates 5hmC modification of these genes by recruiting TET (ten-eleven translocation) protein, and CTCF knockdown affected the protein expression of these genes in vitro.

**Conclusions:**

Our findings demonstrate the increased 5hmC distribution in plasma cfDNA in different disease activity in patients with SLE compared with HCs and relating DhMRs involved in SLE-associated pathways. Furthermore, we identified a panel of SLE relevant biomarkers, and these viewpoints could provide insight into the pathogenesis of SLE.

**WHAT IS ALREADY KNOWN ON THIS TOPIC**

- Growing research suggests that SLE is marked by extensive epigenetic alterations.
- 5-Hydroxymethylome (5hmC), a recently identified altered cytosine variant, is believed to play a crucial role in epigenetic changes during cancer progression, embryonic growth and cellular differentiation.
- The process of DNA methylation has been linked to the development of SLE, yet the role of 5hmC in this mechanism remains largely unexplored.

**WHAT THIS STUDY ADDS**

- This study describes the dynamic changes of 5hmC in plasma cfDNA among patients with SLE with varying disease activities, identifying elevated methylated 5hmC enrichment levels in patients with SLE and preliminarily screening potential biomarkers for diagnosing SLE, uncovering the initial mechanisms through which 5hmC facilitates SLE’s occurrence and progression.

**HOW THIS STUDY MIGHT AFFECT RESEARCH, PRACTICE OR POLICY**

- These results bring a novel insight into 5hmC changes in SLE, identifying 5hmC as candidate biomarkers for the early diagnosis of patients with SLE.
- The application and verification of non-invasive biomarkers in large samples is needed to evaluate the precision and accuracy in diagnosing SLE.
- In-depth analysis of these biomarkers could shed more light on the underlying pathogenesis of SLE, potentially paving the way for more focused therapeutic approaches.

## Introduction

 SLE, a female-predominant autoimmune disease characterised by the production of self-reactive antibodies, hyperactivation of immune cells and loss of immune tolerance, can affect multiple organs in the body, leading to complications, such as nephritis, arthritis and neurological damage.^1^ The early symptoms of SLE are often non-specific, making early diagnosis challenging. The disease follows an unpredictable course, alternating between periods of recurrence and remission. Current treatment strategies can only manage symptoms and delay disease progression but cannot achieve a complete cure.^2^ Accurate early diagnosis is crucial for initiating effective treatments promptly, and biomarkers play a vital role in enhancing the ability to diagnose SLE early. Consequently, developing highly sensitive diagnostic biomarkers for SLE is potentially significant and could substantially impact patient outcomes.

The pathogenesis of SLE has been extensively studied, but the underlying molecular mechanisms have not been fully elucidated. Besides genetic and environmental factors, recent studies have shown that epigenetic alterations, including histone modifications, miRNA dysregulation and DNA methylation, are also widespread in patients with SLE and play significant roles in immune dysfunction, contributing to the disease’s development.^3 4^ 5-Hydroxymethylcytosine (5hmC) is the oxidation product of 5-methylcytosine (5mC) by the ten-eleven translocation (TET) proteins.^5^ It is an important epigenetic mark in the human genome as an intermediate in active DNA demethylation. Recently, increasing evidence has highlighted the crucial role of 5hmC in gene expression regulation and the development of multiple diseases.^6 7^ One study revealed considerable differences in 5hmC levels in the genome-wide promoter regions of CD4+T cells in patients with SLE, suggesting that 5hmC may contribute to the pathogenesis of SLE through abnormal regulation of gene expression. These findings not only indicate that 5hmC is involved in the pathogenesis and disease progression of SLE but also suggest that 5hmC may serve as an ideal molecular biomarker for the occurrence and progression of disease.

Circulating cell-free DNA (cfDNA), small nucleic acid fragmented DNA released into the bloodstream from damaged or ruptured cells, carries genetic and epigenetic information. It can serve as a non-invasive approach for disease diagnosis and prognosis. Recent studies have highlighted the potential of 5hmC signatures in cfDNA as biomarkers for human diseases, suggesting that it could be exploited as a new strategy in disease diagnosis.^8^ In multiple tumours, cfDNA 5hmC signatures have been identified as potential diagnostic biomarkers.^9^,^11^ In type 2 diabetes, cfDNA 5hmC signatures have proven to be biomarkers for diagnosing diabetic retinopathy and nephropathy and for detecting other microvascular complications.^12 13^ In coronary artery disease, cfDNA 5hmC signatures are potential prognostic biomarkers for acute myocardial infarction.^14^ However, the status of 5hmC modifications in cfDNA of patients with SLE and their accuracy and stability as diagnostic biomarkers remain largely unknown. Investigating the presence and potential significance of 5hmC modifications in cfDNA from patients with SLE and their diagnostic value in clinical application is needed.

In this study, we mapped the 5hmC distribution of cfDNA in patients with SLE and HCs using the 5hmC-Seal-seq technique. Our study provides insights into the pathogenesis of SLE and potential valuable biomarkers for SLE diagnosis. Simultaneously, it lays an important foundation for monitoring the occurrence and development of SLE.

## Materials and methods

### Patient characteristics

In total, 35 patients with SLE were recruited from the First Affiliated Hospital of Wenzhou Medical University from 1 June to 31 December 2020. All patients fulfilled the classification criteria of SLE from the American College of Rheumatology and collected blood before therapy. Disease activity was appraised by the SLE Disease Activity Index 2000 (SLEDAI-2K)^15^; patients with SLEDAI scores ≤6 were allocated as stable SLE, and those with >6 were divided as active SLE. Our study comprised 35 patients with SLE and 32 HCs. Online supplemental table 1 provides gender details and SLEDAI-2K scores (mean±SD) of patients with SLE. The cohorts were stratified based on SLEDAI scores into stable SLE, active SLE, validation SLE and HC groups. Each group included two subgroups, and within each subgroup equal amounts of plasma from several patients were pooled together for the extraction cfDNA and creation of a single library. The HC group consisted of individuals without arthralgia, renal or rheumatic diseases. All participants in this study have written informed consent.

### cfDNA 5hmC library construction and sequencing

Ten millilitres of peripheral blood was collected from patients with SLE or HCs. The plasma was prepared by centrifuging the blood at 1600×*g* at 4℃ for 10 min, followed by a second centrifugation step at 16 000×*g* for 10 min. The supernatant plasma was collected and immediately stored at −80°C for future use. Equal amounts of plasma (0.5 mL) from each sample within the same subgroup were pooled (online supplemental table 1), and cfDNA was isolated from the pooled plasma using the QIAamp Circulating Nucleic Acid kit (Qiagen, Valencia, California, USA). 5hmC libraries were constructed as previous reports.^8 16^ First, 15 ng cfDNA was end-repaired and ligated with the Illumina-compatible adaptors. Selective 5hmC chemical labelling was performed by incubating the cfDNA at 37°C for 2 hours in a glycosylation buffer (50 mM HEPES pH 8.0, 25 mM MgCl_2_) containing βGT and N3-UDP-Glc. Then, DBCO-PEG4-Biotin (Click Chemistry Tools, Beijing, China) was added and incubated at 37°C for 2 hours. After the reaction, the biotin-labelled cfDNA was pulled down using C1 Streptavidin beads (Life Technologies, California, USA) by incubating at room temperature for 15 min. Next, the captured cfDNA was amplified through 17 cycles of PCR amplification. The PCR products were purified using 1.0× AMPure XP beads according to the manufacturer’s instruction. The DNA concentration of each library was quantified by Qubit fluorometer (Life Technologies) and quality control performed using a Bio-Fragment Analyser Qseq1. The libraries were sequenced on the Illumina Novaseq 6000 and 150 bp paired-end reads were generated, yielding a minimum of 6G data per library on an S2 flow cell. The sequencing results for patients with SLE and controls were obtained from the same batch, with a common sample identifier (@A00204:644:HKHTWDSXY:1:1:1101:xxxxx:1000). Clean data (clean reads) were generated by removing reads containing adapters, ploy-N regions and low-quality bases from the raw data. Q20, Q30, GC content and number of reads per library were also calculated (online supplemental table 2). All the downstream analyses were performed using the clean, high-quality data.

### Identification of 5hmC modified regions

Clean reads were aligned to the reference genome (ref: hg19) using BWA-MEM V.0.7.12 (online supplemental table 2).^17^ MACS2 (V.2.1.0) was used to identify peak enriched regions.^18^ A q value threshold of 0.05 was regarded as significant enrichment. The chromosome distribution, peak width, enrichment and peak number were displayed. PeakAnnotator was used to identify the 5hmC distribution around the transcription start site (TSS). The distribution of peaks on different function regions, such as 5′UTR, CDS and 3′UTR, was visualised. Homer (V.5.10) was used to detect significantly enriched motif sequences within peaks. The nucleotide tendencies at different positions within the motifs were displayed using sequence logo plots. A motif can be either an exact sequence or a degenerate consensus sequence, allowing for ambiguous characters. A consensus sequence is a string of either nucleotide or protein characters along with ‘degenerate characters’, which specify a subset of possible characters. These degenerate characters can act as ‘wild cards’, representing specific subsets, such as ‘R’ for purines and ‘N’ for any nucleotide.^19^

### 5hmC modified gene dysregulation identification and functional enrichment annotation

Differentially modified genes between samples from patients with SLE and HCs were identified using PePr (V.1.1.18).^20^ Peaks with a read fold change of over 2 were determined as differential and displayed by volcano plot. The location of 5hmC peaks in chromosome displayed by RCircos package in R. To explore the biological connections of the genes with dysregulated 5hmC, functional enrichment annotation analyses including Gene Ontology (GO), Kyoto Encyclopaedia of Genes and Genomes (KEGG) and disease-associated analysis (NHGRI) were performed using KOBAS database for the list of hyper-/hypomethylated 5hmC genes.^21 22^

### Bioinformatics data analyses

According to the sample grouping information, the prcomp function was used for principal component analysis (PCA) analysis and visualised in R. Clustering was performed using the hclust package in R. SLE-associated genes were selected from the DisGeNET database. The hub genes network was generated with the top 10 genes identified with the classifier using the CytoHubba module in Cytoscape based on the betweenness centrality.

### CTCF chromatin immunoprecipitation (ChIP)-sequencing (ChIP-seq)

ChIP-seq was performed to identify the binding site of the transcription factor on the genes. The GSE68978^23^ dataset containing CTCF ChIP-seq of Jurkat and HEK293 and GSE208512/GSE208887/GSE209390^24^ datasets containing CTCF ChIP-seq of primary immune cells were used to analyse CTCF binding sites on candidate genes and visualised by the locally installed Integrated Genome Viewer.

### Cell culture and transfection

The cell line 293T was purchased from the Chinese Academy of Medical Sciences (Shanghai, China) cell bank. The cell was cultured in Dulbecco’s modified Eagle’s medium (Gibco, USA) supplemented with 10% fetal bovine serum (Gibco, USA) and 1% penicillin/streptomycin at 37°C in 5% CO_2_. The siRNAs of CTCF (siRNA1: GTGGTACCATGAAGATGCA; siRNA2: GAACCAACCAGCCCAAACA; siRNA3: CGATTACGCCAGTGTAGAA) were purchased from RiboBio (Guangzhou, China). Transfection was performed using Lipofectamine 2000 reagent (Invitrogen Life Technologies, California, USA) according to the manufacturer’s instructions.

### Western blot

Cells were harvested and lysed in RIPA lysis buffer supplemented with a protease inhibitor cocktail. Antibodies, including BCL, CD83, ETS1 (1:1000, Huabio, China); CTCF (1:1000, Santa Cruz, USA); MYH9 (1:1000, Cell Signaling Technology, USA); TET2 and GAPDH (1:1000, Proteintech, USA), were used. Results were visualised using the Bio-Rad ChemiDoc Touch Imaging System.

### RNA isolation and quantitative real-time PCR (qRT-PCR)

Total RNA was extracted using TRIzol reagent (Thermo Fisher Scientific, California, USA). The extracted RNA (1 µg) underwent reverse transcription using the ReverTraAce qPCR RT Kit (Toyobo, Tokyo, Japan). Real-time PCR was conducted following the manufacturer’s protocol (QIAGEN, 208054) and replicated in triple. Relative mRNA expression was calculated using the comparative threshold cycle (Ct) value and normalised to *GAPDH*.

### 5hmC DNA immunoprecipitation (hMeDIP)-qPCR

hMeDIP-qPCR was performed to detect the 5hmC modification level between negative control (NC) and CTCF knockdown cells. Briefly, the extracted genomic DNA fragmented randomly into 200–1000 bp by ultrasound processes. The hydroxymethylated DNA was then immunoprecipitated using a 5-hmC monoclonal antibody (Diagenode, New Jersey, USA, C15200200-50). Then, the DNA was purified by phenol-chloroform extraction and ethanol precipitation. In qRT-PCR, the dosage of each sample was 1 µL, and input was used as a control. Input correction: % Input=2(Ct_Input_−Ct_ChIP_)×Fd×100%. Here, Fd was the input dilution factor. Primers for hMeDIP-qPCR were listed in online supplemental table 3.

### Gene expression in patients with SLE and HCs

Datasets from the Gene Expression Omnibus (GEO, https://www.ncbi.nlm.nih.gov/geo/) were used for analysis. The GSE81622^25^ dataset included 30 patients with SLE and 25 controls; GSE50772^26^ included 61 patients with SLE and 25 controls; and GSE72326^27^ contained 157 patients with SLE and 20 controls. These datasets were analysed to assess the expression levels of TET1, TET2, TET3 and MYH9.

### Statistical analysis

Continuous data are displayed using mean±SD and compared using the Student’s t-test. Statistical analysis was performed using GraphPad Prism V.8.0.1 software (GraphPad Software, La Jolla, California, USA) and R (V.3.5.0 and 4.2.2). P value <0.05 was regarded as statistically significant.

## Results

### Sample characteristics and cell-free 5hmC-Seal

**Figure 1:**
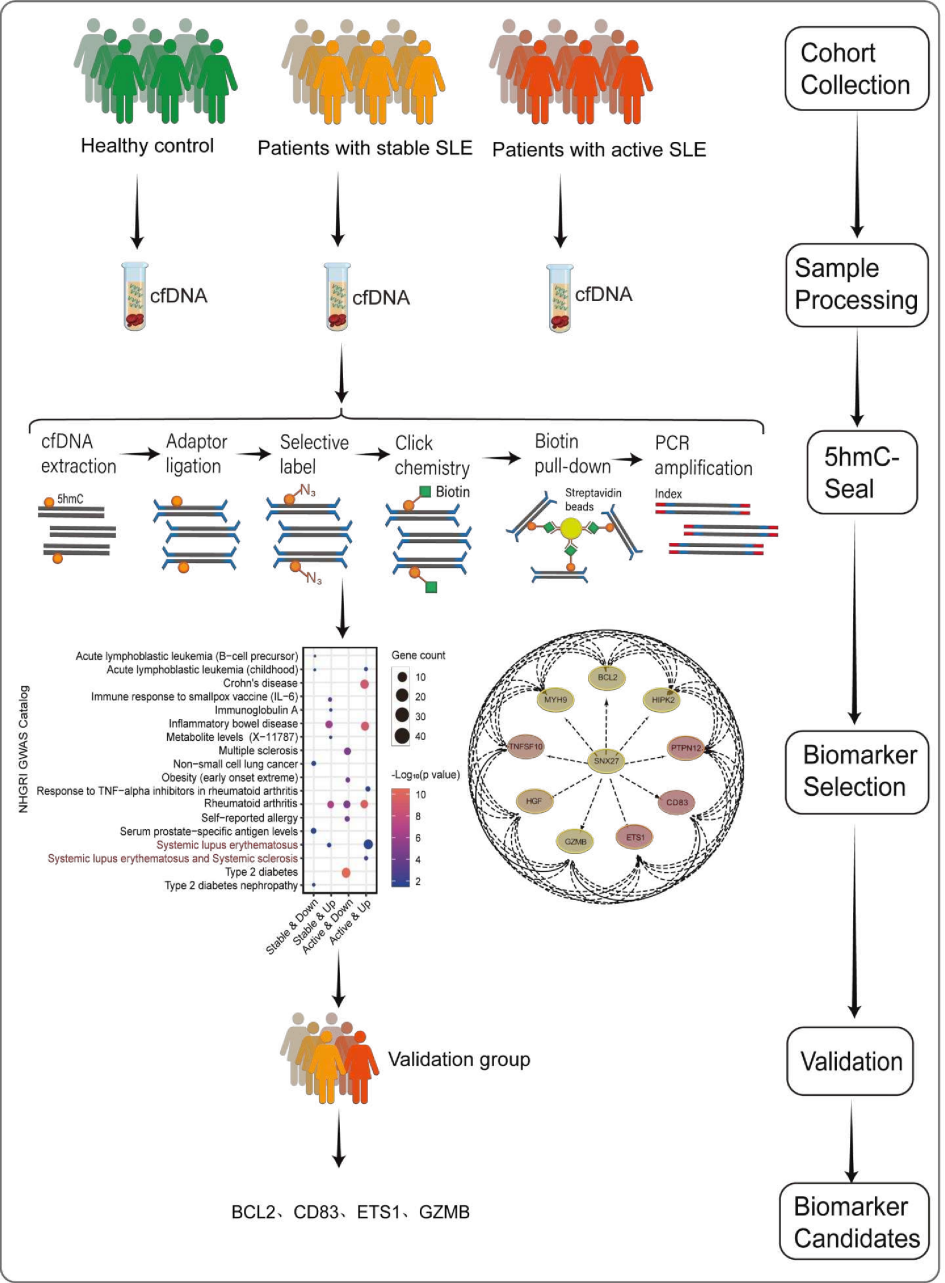
Overview of study design. Plasma cfDNA from patients with SLE was collected during diagnosis. Patients were divided into two groups (stable SLE and active SLE), and a HC group was also included. The workflow of cfDNA 5hmC-Seal profiling is depicted, wherein purified cfDNA was ligated with sequencing adaptors. 5hmC-containing cfDNA fragments were selectively labelled with a biotin group through a two-step reaction. These biotin-labelled cfDNA fragments were captured using streptavidin beads, followed by PCR amplification and next-generation sequencing. DhMGs were screened by bioinformatics analysis, using the validation group SLE to identify candidate biomarkers. 5hmC, 5-hydroxymethylcytosine; cfDNA, cell-free DNA; DhMG, differentially hydroxymethylated gene; HC, healthy control.

A flowchart outlining the analysis of the 5hmC biomarker screen is shown in figure 1. Apart from two male patients in the stable SLE group, all other participants were female (online supplemental table 1). We processed cfDNA from these samples, followed by 5hmC-Seal analysis. Subsequently, potential biomarkers were selected through bioinformatics analysis. These biomarkers underwent further evaluation using the validation group (figure 1).

### cfDNA 5hmC-Seal profiling in HCs and patients with SLE

**Figure 2:**
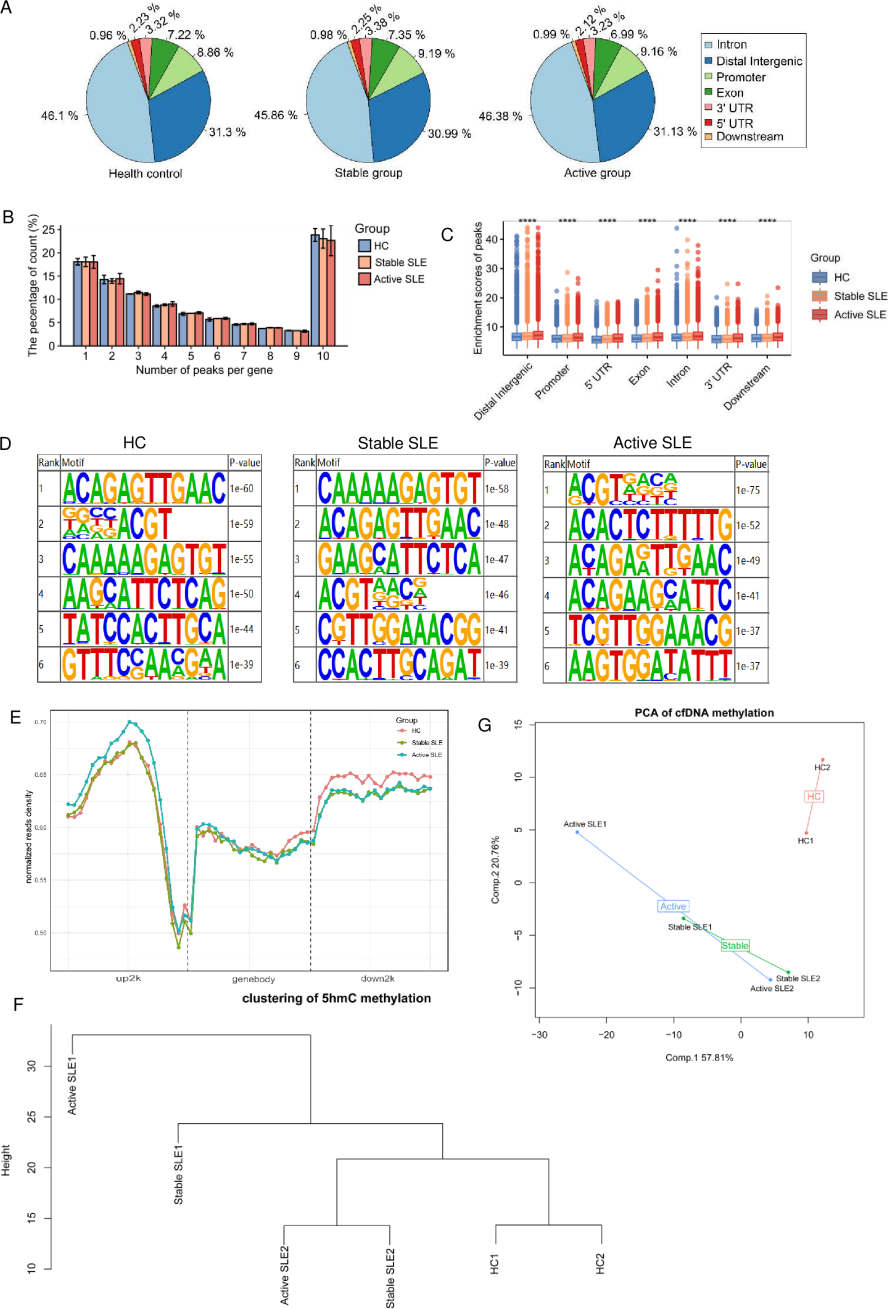
Overall genomic distribution of 5hmC in SLE and HC samples. (A) Pie charts showing the genome-wide distribution of hMRs in plasma cfDNA. (B) Percentage of peak number per gene of cfDNA from HC and SLE samples. (C) Enrichment scores of 5hmC peaks in different genomic features grouped by HC and SLE. (D) Top six enriched motifs detected in hMRs of HC and SLE. (E) Normalised 5hmC read density from HC and SLE samples matched in up2k, down2k and gene body regions. (F) Unsupervised clustering-based 5hmC peaks between HC and SLE. (G) PCA plot of hMRs from HC and SLE samples. 5hmC, 5-hydroxymethylome; cfDNA, cell-free DNA; HC, healthy control; hMR, 5hmC enriched region; PCA, principal component analysis.

We investigated the presence of abnormal 5hmC enrichment in specific regions of cfDNA from patients with SLE and HCs by analysing the 5hmC-enriched regions (hMRs), as illustrated in figure 2A. Notably, in both SLE groups (stable and active) and HCs, the majority of 5hmC localisations were found in introns and distal intergenic areas, followed by promoters and exons, which aligns with findings from a previous study.^28^ To further explore the features of 5hmC enrichment, we counted the number of peaks per gene in stable SLE, active SLE and HC to show the density of 5hmC peaks on genes. Most genes in all three groups had less than 10 peaks detected (figure 2B). Furthermore, we visualised an increasing enrichment of 5hmC in stable SLE and active SLE compared with HC, indicating a correlation between hMRs and the disease activity of SLE (p<0.0001, figure 2C). Transcription factors and proteins, such as histones, bind to DNA with specific preferences rather than randomly. Motif analysis allows us to discern these preferences by analysing DNA sequences for protein-specific binding sites. Through motif enrichment analysis of hMRs, we identified the six most considerably enriched motifs in SLEs and HCs. Figure 2D shows that the top 5hmC motif in stable SLE and active SLE was CAAAAAGAGTGT (p=1e^−58^) and ACGTRRSN (p=1e^−75^), respectively. For HCs, the most predominant 5hmC motif was ACAGAGTTGAAC (p=1e^−60^), followed by DGNBACGT (p=1e^−59^). These findings indicate that the 5hmC motif exhibits specificity within each group, including stable SLE, active SLE and HC.

Next, we analysed the distribution of hMRs in gene bodies, as well as their upstream and downstream regions within 2 kilobases of the gene. Notably, the distribution of 5hmC peaks around upstream 2k regions in the genome of active SLE was considerably higher than HC and stable SLE. Additionally, 5hmC peaks exhibited enrichment both upstream and downstream of the gene body, with lower enrichment observed at the TSS across all groups (figure 2E). Clustering analysis of genome-wide hydroxymethylation revealed differences in the mean 5hmC spectra of plasma cfDNA profiles across the groups. The SLE groups were distinguishable from the HC group, with stable SLE and active SLE showing a closer relationship to each other than to HCs (figure 2F). PCA further validated the results of the cfDNA methylation clustering analysis. Similarly, PCA demonstrated distinct signatures capable of separating SLE groups from HCs, while stable SLEs and active SLE showed partial overlap, suggesting a similarity between these two groups (figure 2G). These findings collectively indicate a significant difference in the 5hmC profile of plasma cfDNA between the SLE groups and HC.

### Functional annotation of deferentially hydroxymethylated regions in cfDNA of stable and active group SLE

**Figure 3:**
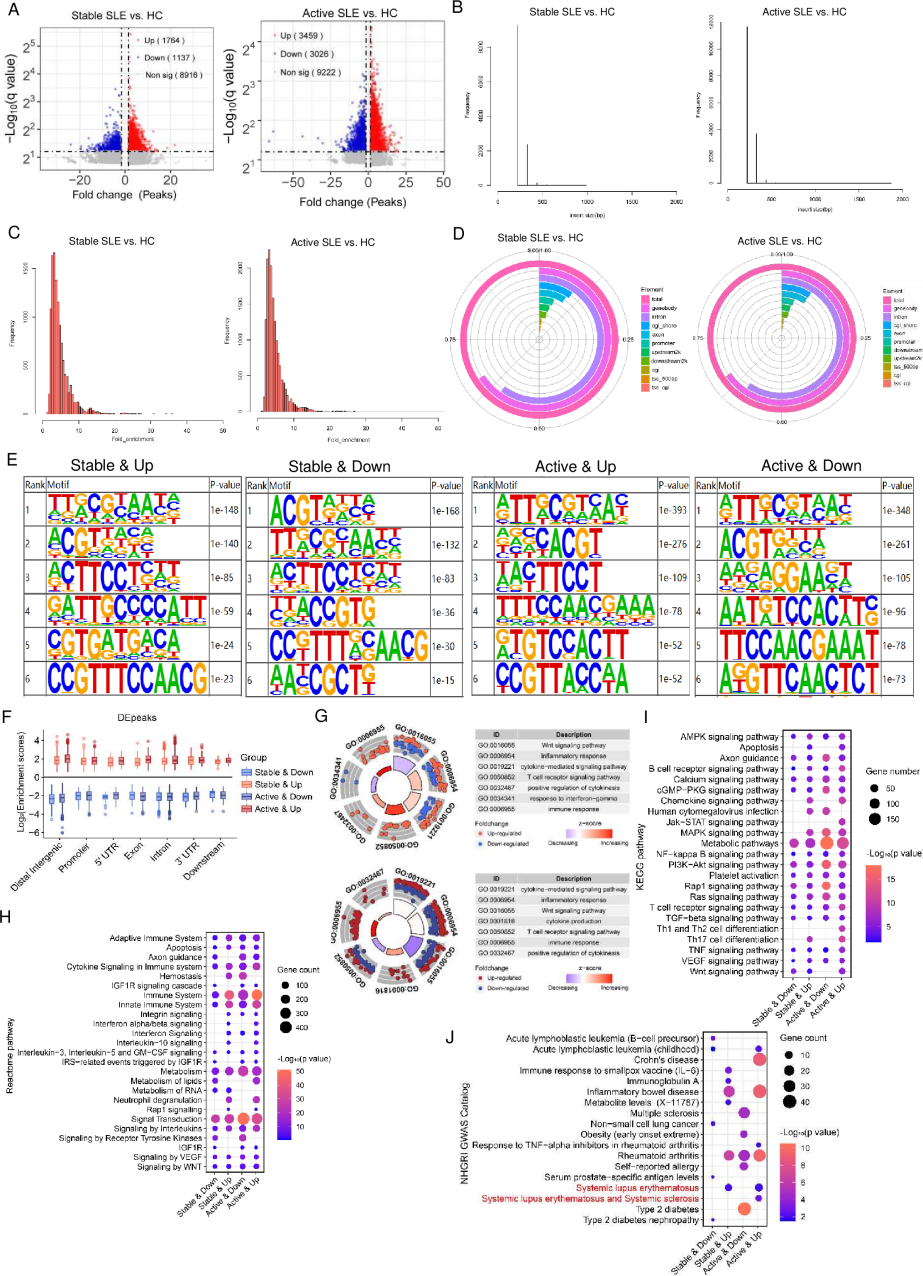
Identification and enrichment analysis of DhMRs in HC and SLE samples. (A) Volcano plot showing significantly 5hmC altered peaks in stable SLE and active SLE compared with HC (|fold change|≥1.5, p value <0.005) highlighted in red (hyper) and blue (hypo). Grey blots represent peaks where differential alterations were not observed. (B) Average frequency of insert size (bp) with DhMRs in stable SLE versus HC (left) and in active SLE versus HC (right). (C) Bar charts displaying the frequency of each fold enrichment of DhMRs in the two groups. (D) Average distribution of DhMR ratios across different genomic regions in stable SLE and active SLE versus HC. (E) Top six 5hmC-modified motif sequences in DhMRs in SLE (stable or active) versus HC. (F) Normalised enrichment score of upregulated or downregulated DhMRs across distinct genomic regions in stable and active SLE relative to HC. (G) Gene Ontology analysis showing the top categories of DhMRs identified from plasma cfDNA from stable SLE versus HC (top) and from active SLE versus HC (bottom). Red blots represent upregulated DhMRs, and blue blots represent downregulated DhMRs. (H, I) Reactome and KEGG pathway analysis of hyper or hypo-DhMRs in stable SLE and active SLE. (J) Pathway analysis of DhMRs in SLE (stable or active) versus HC by NIHGRI GWAS. 5hmC, 5-hydroxymethylome; cfDNA, cell-free DNA; DhMR, differentially hydroxymethylated region; HC, healthy control; KEGG, Kyoto Encyclopaedia of Genes and Genomes.

We performed 5hmC modification differential analysis to explore differences between HCs and patients with SLE. In stable SLE compared with HC, we identified 2901 differentially hydroxymethylated regions (DhMRs), with 1764 up peaks and 1137 down peaks. Similarly, in active SLE compared with HC, we found 6485 DhMRs, with 3459 up peaks and 3026 down peaks (figure 3A). Further, DhMRs were present across all chromosomes, with differing distribution patterns of upregulated or downregulated DhMRs on each chromosome in each SLE group compared with HCs (online supplemental figure 1).

Notably, the frequency of insert size and fold enrichment was considerably higher in active SLE versus HC than in stable SLE versus HC (figure 3B and C). We next analysed the distribution of DhMRs on genome-wide regions in stable SLE and active SLE versus HC. Among all regions examined, the DhMRs were mainly enriched in gene body and introns, with the enrichment rates exhibiting a consistent trend between stable and active SLE versus HC (figure 3D). To comprehensively investigate the motif of 5hmC enrichment in active and stable SLE versus HC, we employed motif enrichment analysis in upregulated and downregulated DhMRs. We found that the motifs of DhMRs were different between each group. Notably, hyper-hydroxymethylated DhMRs were significantly enriched in the TTRCGYAAYN (p=1e^−148^) and ATTGCGTMAY (p=1e^−393^) motifs in stable SLE versus HC and active SLE versus HC, respectively. Conversely, the most enriched motifs in hypo-hydroxymethylated DhMRs were ACGTRDYH (p=1e^−168^) and ATTGCGTMAB (p=1e^−348^) (figure 3E).

Based on these characteristics of DhMRs, we further analysed the relevant enrichment scores and found that the scores of hyper-hydroxymethylation in active SLE versus HC were higher than stable SLE versus HC in each genomic region. Conversely, hypo-hydroxymethylation scores in active SLE versus HC were lower than in stable SLE versus HC, suggesting that significant differences existed in 5hmC enrichment between stable SLE versus HC and active SLE versus HC (figure 3F), with more pronounced changes in 5hmC modification levels as SLE progresses. Overall, these results illustrated that patients with SLE and HC showed differences in both 5hmC enrichment and 5hmC motifs.

Functional enrichment annotation analyses were then performed to further explore the biological process of 5hmC modification involved in SLE. GO analysis revealed that genes with dysregulated 5hmC in both stable and active SLE versus HC were mainly distributed in immune-related functions, including the Wnt signalling pathway, inflammatory response and cytokine-mediated signalling pathway (figure 3G). Additionally, many of these DhMRs were found to participate in crucial pathways, including the immune system, metabolism and signal transduction through the Reactome database (figure 3H). The KEGG analysis similarly indicated that many hyper-DhMRs were enriched apoptosis, chemokine signalling pathway and Th17 cell differentiation (figure 3I). Furthermore, NHGRI GWAS catalogue analysis showed that hyper-DhMRs in cfDNA were related to immune diseases, including Crohn’s disease, inflammatory bowel disease and SLE (figure 3J). Aberrant 5hmC enrichment in promoter and differentially enriched region-related genes could also be relevant to the disease progress. We further performed pathway analysis in their DhMRs. GO results showed that the DhMRs of both stable SLE versus HC and active SLE versus HC were enriched in protein binding, cytoplasm and apoptotic process. Further, KEGG pathways revealed the enrichment in metabolic and immune-related pathways (online supplemental figure 2). Overall, these findings indicated that cfDNA with hyper-DhMRs could be highly associated with SLE, and distinct differences in 5hmC distribution existed during disease progress. This conclusion highlighted the potential of 5hmC as a candidate biomarker for the diagnosis and monitoring of SLE.

### 5-hydroxymethylcytosine features and functional annotation in validation group SLE

Expanding on our previous findings indicating higher hyper-DhMRs in patients with SLE, we included a group of patients with SLE with higher SLEDAI scores to investigate the association between 5hmC and disease progression. This subgroup exhibited higher frequency of insert size and fold enrichment compared with the active SLE versus HC group (online supplemental figure 3A and online supplemental figure 3B). The increased frequency in this subgroup confirmed that the 5hmC levels rise with SLE activity.

Since more than half of the hyper-hMRs in both stable SLE versus HC and active SLE versus HC were located in gene bodies, we analysed the genome-wide distribution of hyper-hMRs in the validation group SLE (online supplemental figure 3C). We also analysed the motifs of hMR-enriched regions in the validation group SLE, with YACGTRNH (p=1e^−391^) ranking first in hyper-hMRs and YACGTRNH (p=1e^−495^) in hypo-hMRs (online supplemental figure 3D), which was different from the observation in stable SLE versus HC and active SLE versus HC, suggesting that the 5hmC enrichment motifs had different preferences in different disease activity states. Moreover, we identified up to 29 527 DhMRs in the validation group SLE, including 8796 up and 20 731 down, which was substantially higher than observed in stable SLE versus HC and active SLE versus HC (online supplemental figure 3E). Similarly, we analysed the distribution of DhMRs in chromosomes (as shown in online supplemental figure 3F).

We conducted GO and KEGG function annotation analyses on the promoter and differentially enriched region-related genes to elucidate the biological significance of DhMRs in the validation group SLE versus HC. GO analysis showed that these DhMRs in promoter and differentially enriched region-related genes were notably enriched in protein binding, immune response and signal transduction. Further, KEGG functional enrichment analysis showed that many DhMRs in promoters were enriched in metabolic pathway. Additionally, a higher concentration of DhMRs existed from differentially enriched region-related genes in pathways such as the cAMP signalling pathway, regulation of actin cytoskeleton, Ras signalling pathway and Rap1 signalling pathway (online supplemental figure 4).

### Screened DhMGs as a candidate biomarker to distinguish patients with SLE and HC

**Figure 4:**
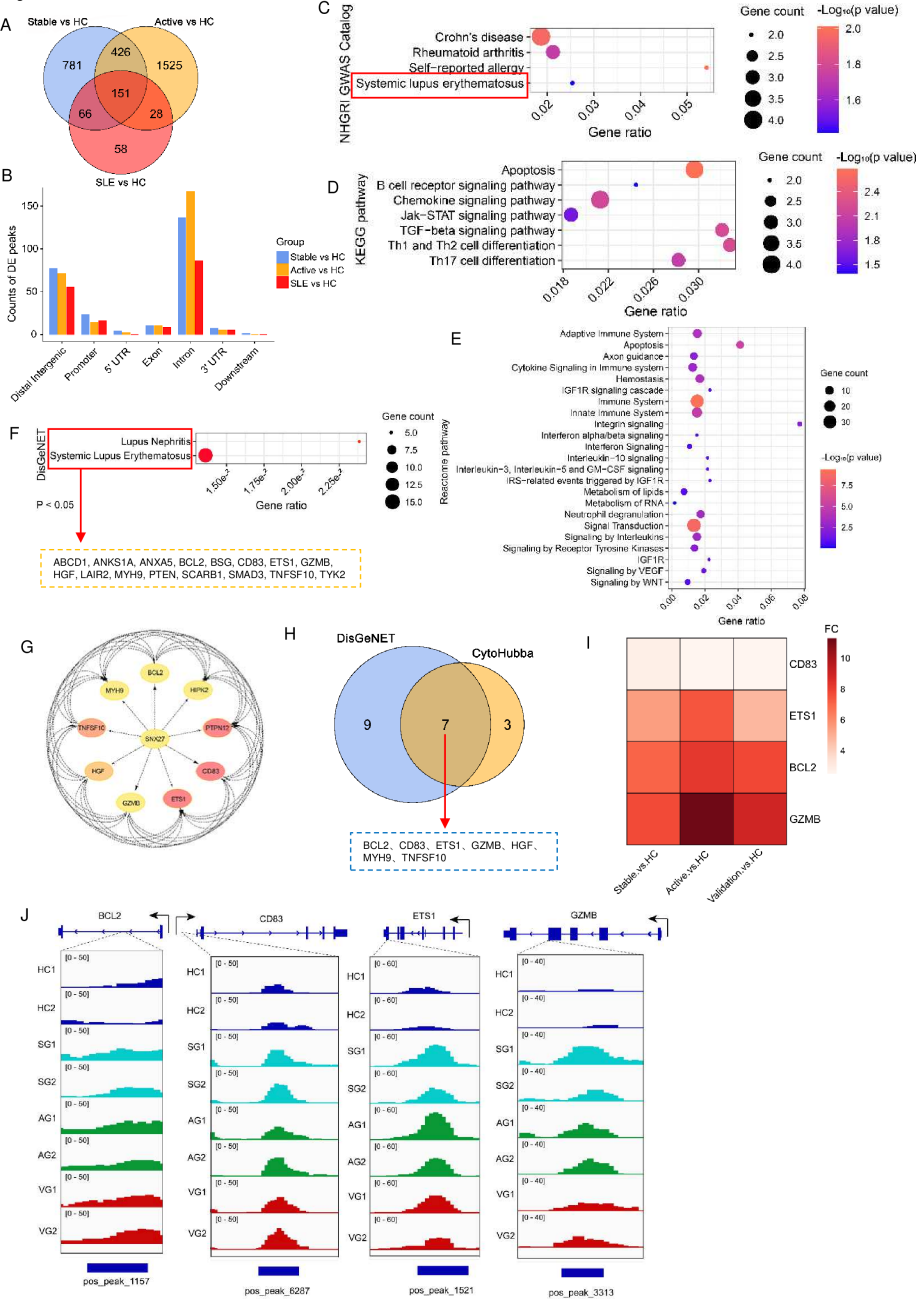
Screening for potential diagnostic biomarkers of SLE based on DhMGs in plasma cfDNA. (A) Venn diagram showing the overlap between significantly expressed hyper-DhMRs in cfDNA from stable SLE versus HC, active SLE versus HC, and SLE (stable+active SLE) versus HC. (B) Distribution of counts across different genomic regions of 151 hyper-DhMGs. (C) Pathway analysis of the 151 overlapping hyper-DhMGs by NIHGRI GWAS. The red box shows the enrichment of these DhMGs in SLE. (D, E) DhMGs (151) were grouped in functional pathways by the KEGG and Reactome analyses. (F) DhMRs were obviously enriched in SLE and lupus nephritis (p<0.05) according to the DisGeNET analysis. The dashed box shows 16 out of 151 DhMGs gathered in these pathways. (G) Hub genes (10/151) in DhMGs were identified by CytoHubba. (H) Venn diagram showing the overlap of DhMGs from the DisGeNET and CytoHubba analyses. (I) Heatmap showing hyper-5hmC genes in stable SLE versus HC, active SLE, and validation SLE versus HC. (J) Representative genome browser views of 5hmC signals in different SLE groups and HC. Genome browser views of 5hmC amplifications detected in BCL2, CD83, ETS1 and GZMB gene regions formed libraries generated with plasma cfDNA from the stable SLE (SG), active SLE (AG), validation SLE (VG) and HC groups. 5hmC, 5-hydroxymethylome; cfDNA, cell-free DNA; DhMGs, hyper-differential hydroxymethylation genes; HC, healthy control; KEGG, Kyoto Encyclopaedia of Genes and Genomes.

Our results strongly suggest that hyper-DhMRs are closely associated with biological processes and immune regulation relevant to SLE. We conducted further analysis on hyper-differential hydroxymethylation genes (DhMGs) in stable SLE versus HC, active SLE versus HC, and SLE (stable SLE+active SLE) versus HC. Compared with HC, we identified 151 overlapping DhMGs with hyper-5hmC in distinct SLE activity groups (figure 4A). These DhMGs were predominantly enriched in intron and distal intergenic regions, followed by promoter and exon regions (figure 4B). NHGRI GWAS catalogue analysis revealed enrichment of these 151 DhMGs in SLE, rheumatoid arthritis (RA) and Crohn’s disease (figure 4C). Further, KEGG analysis indicated involvement in pathways related to cell apoptosis, chemokine signalling pathways and cell differentiation (figure 4D), indicating a potential role for 5hmC modification in mediating immune cell differentiation and contributing to SLE progression. Similarly, Reactome pathway analysis highlighted pathways such as the immune system, innate immune system and signal transduction (figure 4E).

To further identify potential biomarkers of SLE within these DhMRs, we initially identified 16 out of 151 DhMGs with hyper-5hmC enrichment in SLE and lupus nephritis based on DisGeNET analysis (figure 4F). Moreover, 10 hub DhMGs were obtained through CytoHubba analysis, and their interactions were visualised (in figure 4G). By combining these two screening methods using a Venn plot, we identified seven common DhMGs (figure 4H). To further validate these seven key candidate DhMGs’ 5hmC modification levels, a validation group was used. Results showed that *CD83*, *ETS1*, *BCL2* and *GZMB* genes also exhibited upregulated 5hmC enrichment in validation SLE versus HC (figure 4I and J). This further suggested that CD83, ETS1, BCL2 and GZMB may be closely linked to SLE progression and have the potential to serve as biomarkers. Furthermore, PCA of cfDNA 5hmC profiles demonstrated a clear distinction between HCs and SLEs. Validation SLEs could be distinguished from stable SLEs and active SLEs, although the latter two clustered closely, with less apparent differences (online supplemental figure 5).

### CTCF-mediated DNA hydroxymethylation on the regulation of candidate biomarkers in vitro

**Figure 5:**
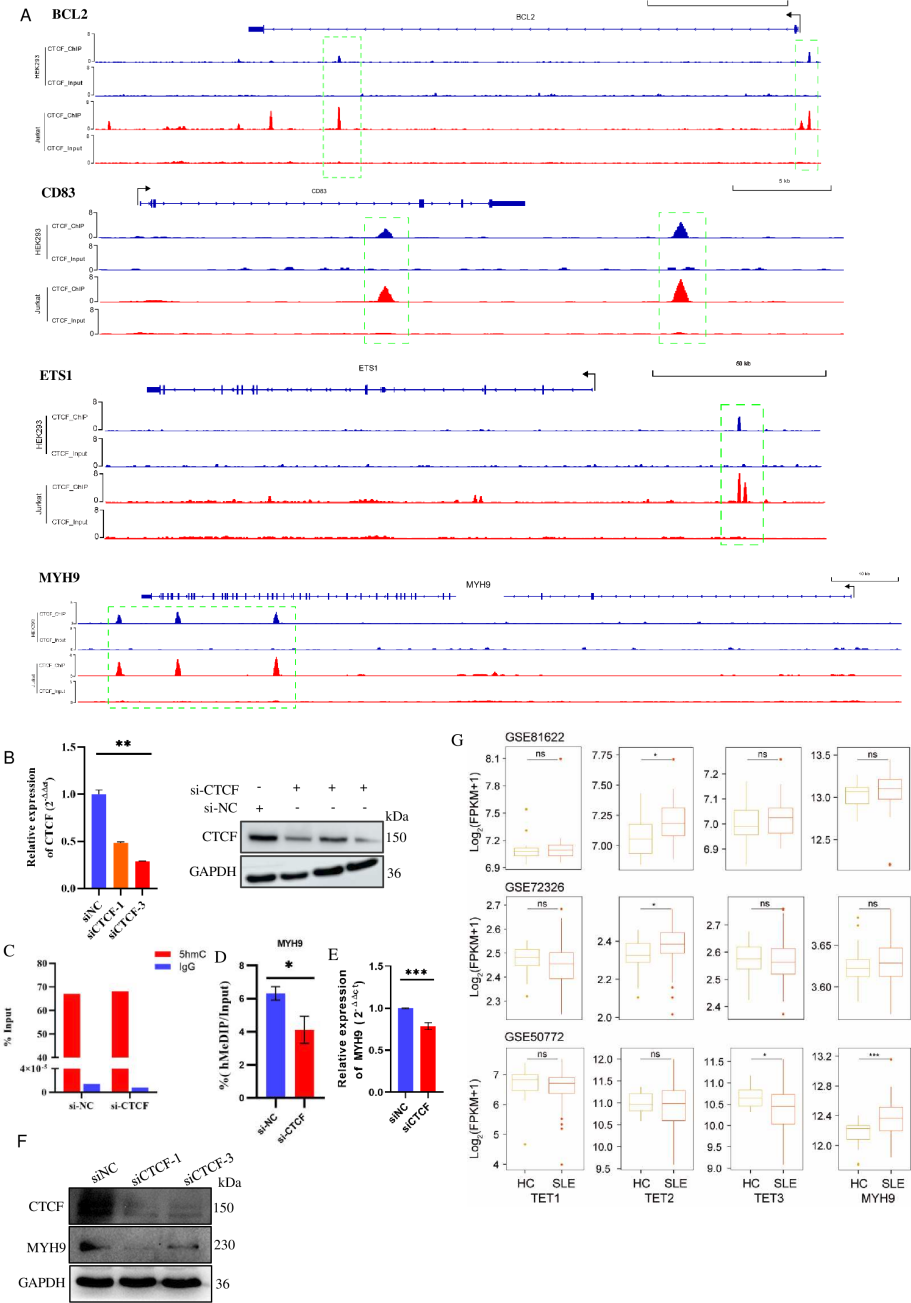
CTCF regulates the DNA hydroxymethylation level and gene expression of candidate DhMGs. (A) Binding regions of the transcription factor CTCF in BCL2, CD83, ETS1 and MYH9 of HEK293 and Jurkat cell lines. (B) CTCF mRNA and protein levels in 293T cells transfected with CTCF-siRNA or negative control (NC). GAPDH was used as an internal control. (C) Enrichment efficiency of anti-5hmC antibody and IgG antibody in si-NC and si-CTCF groups evaluated using a 5hmC-positive external reference in vitro via hMeDIP-qPCR. (D) 5hmC modification level of MYH9 (hMeDIP/Input) in 293T cells transfected with CTCF-siRNA or si-Control. (E, F) MYH9 mRNA and protein levels in 293T cells transfected with CTCF-siRNA. (G) Relative TET1, TET2, TET3 and MYH9 expression (FPKM) in SLE and HC, as analysed using the GSE81622, GSE72326 and GSE50772 datasets. ^*^p<0.05; ^***^p<0.001; ns, no significant difference. 5hmC, 5-hydroxymethylome; DhMG, hyper-differential hydroxymethylation gene; HC, healthy control.

To explore whether these candidate biomarkers screened in the previous step play a role in CTCF-mediated DNA hydroxymethylation, we downloaded the ChIP-seq data for CTCF in HEK293 cells, Jurkat cells and primary immune cells from public databases. We verified the presence of CTCF binding sites in the DNA sequences of the seven candidate genes. CTCF binding sites were detected in *BCL2* (promoter, gene body), *CD83* (gene body, 3′ UTR), *ETS1* (promoter) and *MYH9* (gene body, 3′ UTR) across these analysed cell lines (figure 5A and online supplemental figure 7). However, no CTCF binding sites were detected in the DNA sequences of *GZMB*, *HGF* or *TNFSF10* (online supplemental figures 6,7). These findings suggest that CTCF-mediated 5hmC modification may play a regulatory role in BCL2, CD83, ETS1 and MYH9, but not influence 5hmC of the latter three genes in vitro.

By testing at both the RNA and the protein levels, the results showed that we successfully obtained CTCF knockdown 293T cells by transfecting CTCF-siRNA (figure 5B). Next, hMeDIP-qPCR was used to verify the effect of CTCF knockdown on the 5hmC enrichment level of candidate DhMGs in the 293T cell. Positive internal reference (5hmC-modified DNA) was added to the sample to be tested in vitro, and the results showed that hMeDIP successfully enriched 5hmC-modified DNA in this experiment (figure 5C). Then, the hMeDIP-qPCR was performed, and the DNA hydroxymethylation level of *MYH9* was considerably reduced after CTCF knockdown compared with the NC (figure 5D). Additionally, the mRNA and protein expression levels were substantially downregulated after CTCF knockdown compared with the NC (figures 5E,F). In contrast, the DNA hydroxymethylation levels of *BCL2*, *CD83* and *ETS1* were increased with CTCF knockdown (online supplemental figure 8A), indicating the mechanism by which 5hmC exerts their effects on these DhMGs may not only be directly mediated by CTCF recruiting TET, but also involves interactions with other factors.

To further explore the role of TET proteins in SLE, we analysed their expression levels in the GSE81622, GSE72326 and GSE50772 datasets. Our analysis revealed that TET2 was significantly upregulated in SLE (GSE81622 and GSE72326 datasets; p<0.05). Conversely, TET3 was significantly downregulated in SLE (only in the GSE50772 dataset; p<0.05), whereas TET1 showed no statistical difference in expression between SLE and HC across all three datasets (figure 5G). This suggests that TET2 may play a potentially important role in 5hmC modification in SLE.

To investigate whether DhMGs with 5hmC modification are regulated by CTCF-mediated TET2 enzyme, we used western blotting to detect the TET2 protein expression alterations with CTCF knockdown. Results showed that CTCF knockdown did not affect TET2 protein expression, suggesting that CTCF does not regulate the 5hmC modification of these DhMGs through the regulation of TET2 protein expression (online supplemental figure 8B). The specific mechanism is complex and requires further exploration. Subsequently, the protein expression of these DhMGs was also examined with CTCF knockdown. The results showed that BCL2 expression evidently increased, while CD83 expression decreased, and ETS1 expression remained consistent with the NC (online supplemental figure 8B). This indicates that these gene expressions were also affected by CTCF knockdown in vitro.

In summary, we found that both 5hmC modification and expression levels of MYH9 decreased after CTCF knockdown. Furthermore, we observed a trend toward upregulated MYH9 expression in patients with SLE using the GSE81622 and GSE72326 datasets, with significant upregulation observed in the GSE50772 dataset (figure 5G). This suggests that MYH9 may be directly affected by CTCF-recruited TET enzyme-mediated DNA hydroxymethylation in vitro. These results imply that MYH9 may play a crucial role as a DhMG in the development of SLE and could serve as a potential diagnostic biomarker for the disease.

## Discussion

### New strategies for diagnosis and treatment of SLE

SLE is a genetically predisposed disease primarily affecting women. Its pathogenesis involves various factors such as genetics, epigenetics, molecular processes, immunomodulation and environmental influences. Patients affected by SLE often exhibit increased apoptosis, defective clearance of late apoptotic debris, heightened exposure to autoantigens, abnormal immune responses and production of autoantibodies. Clinical manifestations vary widely due to gender and age differences among patients, with systemic involvement presenting diverse and non-specific symptoms.

Currently, SLE diagnosis relies on clinical manifestations and laboratory tests. However, early-stage rates are limited, often leading to delayed treatment and disease progression due to atypical symptoms in many patients. Identifying sensitive diagnostic markers is crucial to enhance early diagnosis, enabling timely intervention and treatment to control the disease and reduce relapses.

Elevated levels of cfDNA have been observed in rheumatic disease due to key cell death processes like apoptosis and necrosis.^29^ Damaged cells release fragmented DNA into the plasma, and changes in cfDNA concentration in peripheral blood can reflect disease progression, offering potential diagnostic insights. Epigenetic modification is increasingly recognised as a pivotal mechanism in SLE, involving DNA methylation, histone modification and non-coding RNA-mediated gene regulation. Studies have identified DNA hypomethylation as a key molecular mechanism in SLE, with specific gene methylation levels serving as sensitive diagnostic markers, distinguishing patients with SLE from healthy individuals and other autoimmune diseases.^30^

5hmC, a stable intermediate product of active DNA demethylation, regulates gene expression and plays a vital role in gene expression regulation. It is closely associated with disease occurrence and development. Integrating the cfDNA 5hmC profile into multi-omics analyses enhances disease detection sensitivity,^31^ offering a promising avenue for non-invasive and highly sensitive disease diagnosis. Our study explores whether the 5hmC alteration signature of cfDNA in patients with SLE can serve as biomarkers for non-invasive SLE diagnosis, aiming to improve early detection efficiency.

In this study, we employed the 5hmC-Seal technique to map the genome-wide 5hmC modification profiles in circulating cfDNA from patients with SLE with varying disease activities and HCs. Our findings reveal that genes with hyper-5hmC were highly associated with SLE disease occurrence and progression. Additionally, through bioinformatics analysis, we identified potential 5hmC-related DhMGs and further elucidated the possible molecular mechanism of these abnormal DNA hydroxymethylation biomarkers involved in regulating the pathogenesis of SLE. Our study is the first to report that differential 5hmC signatures of cfDNA can distinguish patients with SLE from HCs and provide potential biomarkers for early diagnosis and treatment of SLE. However, a key limitation of the study must be acknowledged. cfDNA originates from various cell types, meaning that differences in cell subset composition between patients and controls could significantly impact our findings. This variability poses a challenge, as it would have been more accurate to adjust for these differences in cell subset composition before conducting the analysis. Considering the high heterogeneity of SLE, further large-scale clinical studies are needed to validate the sensitivity and specificity of cfDNA 5hmC signatures in SLE diagnosis, facilitating the transition of this potential biomarker into the clinical setting.

### 5hmC signature alterations in cfDNA with different SLE disease states

Recent studies have found that 5hmC signature alterations are closely related to gene expression regulation, pluripotent stem cell differentiation, neuronal development and tumourigenesis.^32^ Human diseases can lead to 5hmC alterations in cfDNA. Studies have shown that the 5hmC modification in tumours is considerably different from that in normal tissues, and cfDNA released by different tumours has different 5hmC signatures, suggesting its potential as a candidate target for non-invasive liquid biopsy.

In a large covariate-controlled study testing a 5hmC-based classifier to detect colorectal cancer, cfDNA proved to be an excellent predictor of CRC. The inclusion of 5-hmC in multianalyte testing could improve sensitivity for the detection of early-stage cancer, surpassing conventional detection methods.^33^ Compared with HC, the 5hmC modifications in cfDNA of non-small cell lung cancer patients exhibit distinctly 5hmC gains in both gene bodies and promoters. Furthermore, confirmed 5hmC-related candidate biomarkers provide potentially valuable biomarkers for non-invasive diagnosis of non-small cell lung cancer.^10^

In addition to tumours, 5hmC of cfDNA reveals insight into autoimmune disease regulation. Hydrogen sulfide promotes TET1 and TET2 expression, which are recruited to Foxp3 by TGF-β and IL-2 signalling to maintain Foxp3 5hmC methylation and Treg cell-associated immune homeostasis.^34^ Inhibiting miR-21 could reduce intracellular iron accumulation and DNA hydroxymethylation in CD4^+^ T cells, which contributed to improving organ damage and disease progression in MRL/lpr mice.^35^ Increased 5hmC in CD4+T cells suggests that DNA hydroxymethylation contributes to the aberrant regulation of gene transcription in the pathogenesis of SLE.^36^ Our research also indicates that 5hmC alterations in SLE cfDNA are widely occurring. We described genome-wide regions differential modification patterns of cfDNA 5hmC in SLE compared with HC and obtained the SLE-related 5hmC biomarkers in cfDNA and preliminary disclosure of their possible molecular mechanisms involved in regulating the pathogenesis of SLE. These findings point to the significance of epigenetic mark 5hmC as a potential biomarker or promising therapeutic target, and the integration of cfDNA 5hmC profiles with multi-omics analysis can enhance the sensitivity of disease detection, offering a new direction for non-invasive disease screening and diagnosis.^31^ Additionally, in diseases other than tumours, the 5hmC modification changes in cfDNA may also have potential diagnostic and therapeutic value, making it a valuable direction for future research.

### SLE disease biomarker screening

Numerous studies have reported an association between 5hmC and autoimmune diseases, such as RA^7^ and multiple sclerosis,^37^ suggesting a crucial role of 5hmC in autoimmune diseases. However, the impact of DNA 5hmC in cfDNA on the development of SLE remains poorly understood. Therefore, the current analysis of 5hmC profiles of cfDNA in SLE is pivotal for advancing our understanding of the disease. We present differential expression patterns of 5hmC across genome-wide regions in SLE cfDNA compared with HC. We found that cfDNA 5hmC peaks were gathered in intron and distal intergenic of gene bodies in both SLEs and HC. These were consistent with the previous study reported in lung cancer.^10^ At up2k of gene body, the reads density of active SLE was higher than stable SLE and HC, suggesting that an increase in 5hmC modification might predict an increase in the severity of disease in patients with SLE. The unsupervised clustering analysis based on 5hmC revealed that SLEs and HCs exhibited indistinct clustering patterns, highlighting significant disparities in the localisation of 5hmC between these two groups. Similarly, the unbiased PCA also identified distinct signatures capable of differentiating SLEs from HCs. However, both the clustering analysis and the PCA failed to distinguish stable and active SLEs, which could potentially be attributed to the limited number of samples in our study. Next, we screened 151 DhMGs with hyper-regulated in cfDNA of SLE, which were enriched in SLE-related pathways. Seven candidate biomarkers related to the occurrence and development of SLE were proposed through further screening. Moreover, further detection showed that with the knockdown of CTCF, the 5hmC modification level and expression level of MYH9 were downregulated. MYH9 encodes the heavy chain of non-muscle myosin IIA, a widely expressed cytoplasmic myosin participating in visceral endoderm cell-cell adhesion and placenta formation.^38^ MYH9 has been found to influence lots of autoimmune diseases, including MYH9-related platelet disorders,^39^ RA,^40^ primary membranous nephropathy^41^ and lupus nephritis.^42^ However, the role of differential methylation of MYH9 in diseases has rarely been reported, especially in SLE. Only one study found that MYH9 was shown to upregulate gene expression and demonstrated hypomethylation in the acute and chronic resistance exercise.^43^ Our results identify that MYH9 could act as a DhMG, providing new insights into DNA hydroxymethylation in SLE.

### Potential regulatory mechanisms of 5hmC in SLE

In SLE, the specific mechanisms of 5hmC modification regulation have not been fully elucidated. Therefore, we explored the mechanisms of 5hmC modification regulation in the abovementioned candidate DhMGs through in vitro experiments. Many DhMRs were enriched in introns and intergenic regions often associated with H3K4me1 histone markers, promoter flanking regions and CTCF binding sites.^44^ As a transcription factor, CTCF can regulate promoter and enhancer activities, and chromatin structure.^45 46^ Furthermore, CTCF can promote DNA hydromethylation by recruiting TET enzymes, which enzymatically convert 5mC to 5hmC. For example, CTCF recruits and interacts with TET enzymes to promote DNA hydroxymethylation in the lipogenesis transcriptional enhancer.^47^ Additionally, previous studies have reported that in SLE CD4^+^ T cells, the transcription factor CTCF may regulate the expression of the *SOCS1* gene by modulating the hydroxymethylation of its promoter.^36^ Therefore, in this study, CTCF ChIP-seq was used, and it found that CTCF can bind to different regions of the *BCL2*, *CD83*, *ETS1* and *MYH9*, indicating that these genes may exist with 5hmC modification and are regulated via TET recruited by CTCF. We further use hMeDIP-qPCR analysis to confirm that these genes do exist with 5hmC modifications, but only 5hmC modification of the *MYH9* has been downregulated after CTCF is knocked down. The remaining three genes have been upregulated, suggesting that *MYH9* may be the target gene for CTCF recruiting TET-mediated, but the other three genes are not regulating their 5hmC modification through this mechanism. In theory, CTCF binding to low methylated regions could mediate local DNA demethylation through TET recruitment.^48^ Moreover, the distal CTCF binding site of the TET1 and TET2 gene promoters acts as an enhancer, and high CTCF occupancy in the stronger regions of TET1 and TET2 induces their high expression in iAs-transformed cells.^49^ However, our experiment found that CTCF knockdown did not affect the TET2 protein expression, indicating that CTCF may mediate the 5hmC modification of these three genes by influencing unknown demethylase. Further research is needed to elucidate the specific mechanisms of the interaction between CTCF and DNA demethylases in SLE.

Following CTCF knockdown, the protein levels of these four DhMGs were observed, and MYH9 protein expression decreased with the modification of 5hmC, which further indicated that *MYH9* was a 5hmC-mediated target gene directly regulated by CTCF recruiting TET. However, the other three candidate genes with 5hmC modification were upregulated, but the changes in gene expression levels were not necessarily consistent. One possible reason is that the gene expression level is often associated with its 5hmC modification location in the genome. The 5hmC content in the gene body is often consistent with gene expression activity, which can promote or maintain gene expression.^50^ Similarly, the promoter region 5hmC content was significantly positively correlated with transcription levels, and this relationship was partially dependent on CpG density in the promoter.^51^ However, the level of 5hmC in the genome is not a simple linear relationship to high gene expression. For example, one research found that the 5hmC level is very low for both the promoter and the gene body of a set of genes with constantly high expression levels, such as housekeeping genes.^52^ These highlighted that the regulatory effect of gene expression by 5hmC may be different due to the distribution of 5hmC in the genome. Moreover, some of the effects observed in CTCF knockdown could be due to disruptions in chromatin looping as CTCF plays a crucial role in maintaining chromatin architecture, thereby influencing both cis and trans regulatory mechanisms.^53^ Other post-transcriptional regulation factors may also play a role in the gene expression besides DNA hydroxymethylation. For example, a study revealed the dynamic reprogramming of DNA 5hmC and RNA m^5^C during the embryonic organogenesis after implantation, illustrating that DNA modification and RNA methylation can be co-regulated in time and space through the transcriptome, forming a well-coordinated network.^54^ In conclusion, all these may cause inconsistent protein expression of DhMGs after CTCF knockdown. Therefore, further mechanisms should be explored in the future.

## Conclusions

In our study, we observed elevated levels of plasma cfDNA genome-wide 5hmC in patients with SLE with different disease activity compared with HCs. This increase was accompanied by upregulated expression of TET2 in SLE, which converts 5mC to 5hmC. While this may partially explain the hyper-5hmC modification in SLE cfDNA, complete differentiation of patients with SLE based on 5hmC levels alone remains challenging due to the limited sample size. Further analysis identified *MYH9* as a potential molecular diagnostic marker for SLE development. However, the mechanism behind increased hydroxymethylation of genes is not fully explained by elevated TET expression alone. We further explored this mechanism and demonstrated that CTCF, a transcription factor, can mediate DNA hydroxymethylation and regulate DhMGs expression in vitro. In summary, our study reveals key genome-wide 5hmC differences in SLE cfDNA and identifies new potential biomarkers for the diagnosis and treatment of SLE, providing a further understanding of SLE pathogenesis.

## supplementary material

10.1136/lupus-2024-001286 online supplemental file 1

## Data Availability

Data are available in a public, open access repository.
