# Supplementary material for: 5-Hydroxymethylcytosine in circulating cell-free DNA as a potential diagnostic biomarker for SLE
Source: Lupus Sci Med. 2024 Oct 4;11(2):e001286. doi: 10.1136/lupus-2024-001286 (PMC11459320; doi:10.1136/lupus-2024-001286)
Supplement: online supplemental file 1 [file lupus-11-2-s001.pdf]

Stable SLE vs HC

A

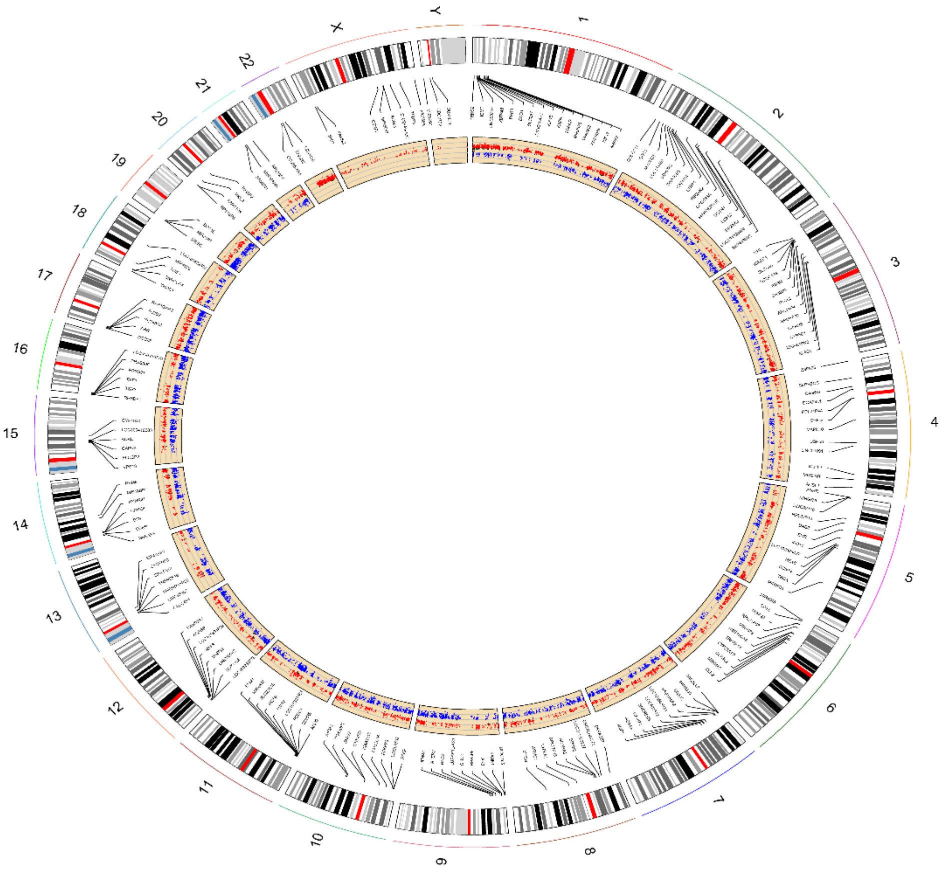

Active SLE vs HC

B

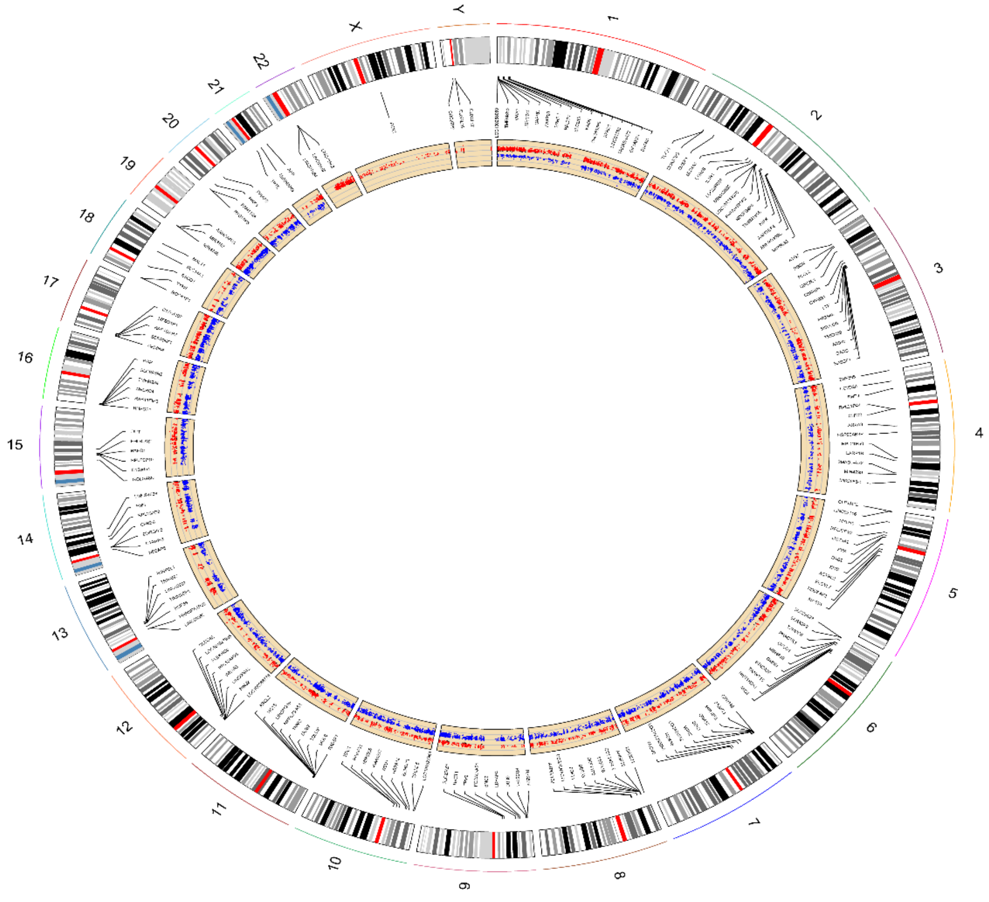

Supplementary Figure 1 Landscape of DhMRs in chromosomes. (A and B) Distribution of DhMRs in the chromosomes of stable SLE vs. HC (A) and active SLE vs. HC (B). The circle presents a chromosome map, and the numbers outside the circle indicate the chromosome number. Different colors were used to distinguish hyper (red) and hypo (blue) DhMRs.

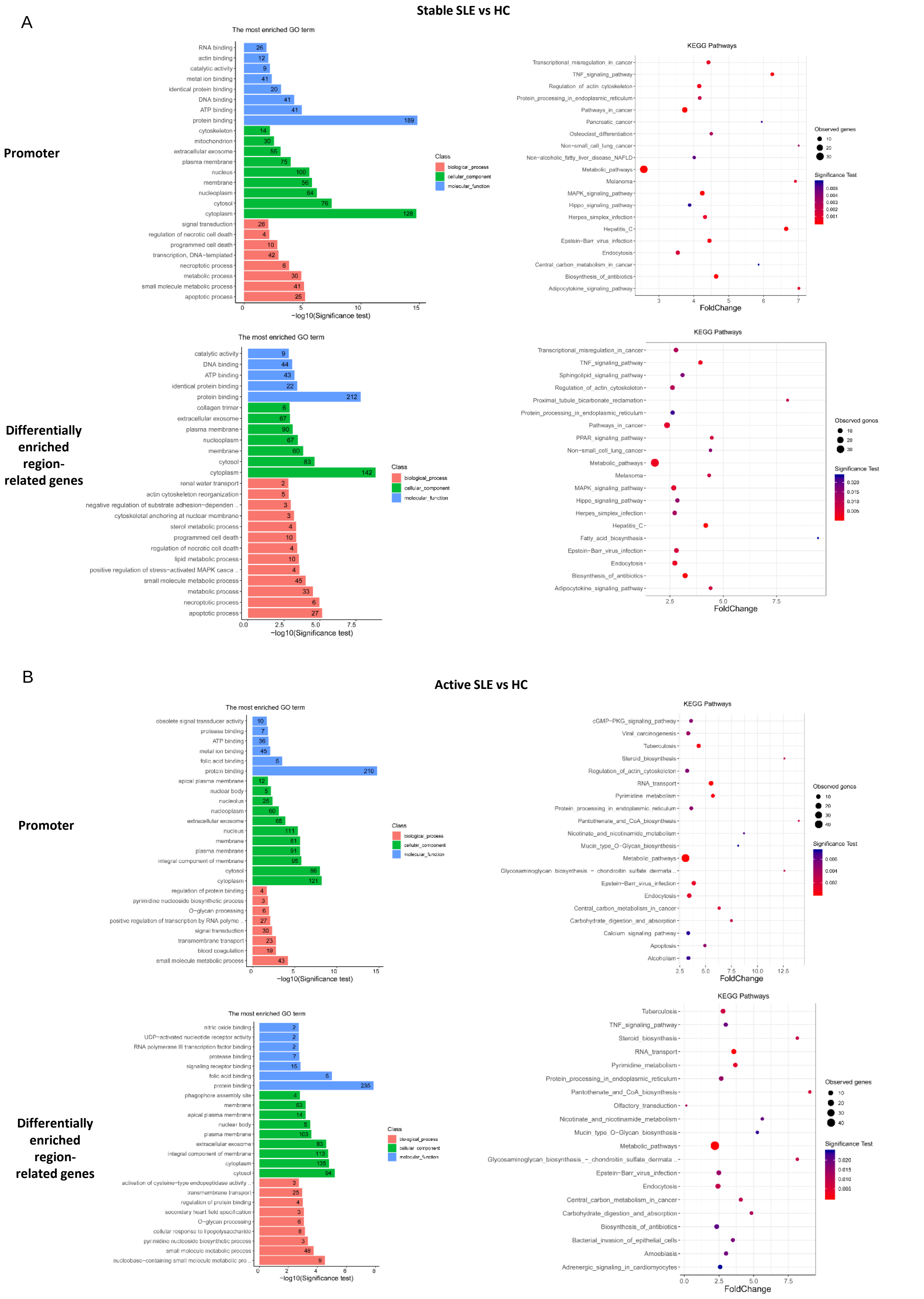

A
Stable SLE vs HC
Promoter
Differentially enriched region-related genes
The most enriched GO term
RNA binding
actin binding
catalytic activity
metal ion binding
identical protein binding
DNA binding
ATP binding
protein binding
cytoskeleton
mitochondrion
extracellular exosome
plasma membrane
nucleus
membrane
nucleoplasm
cytosol
cytoplasm
signal transduction
regulation of necrotic cell death
programmed cell death
transcription, DNA−templated
necroptotic process
metabolic process
small molecule metabolic process
apoptotic process
−log10(Significance test)
Class
biological_process
cellular_component
molecular_function
KEGG Pathways
Transcriptional_misregulation_in_cancer
TNF_signaling_pathway
Regulation_of_actin_cytoskeleton
Protein_processing_in_endoplasmic_reticulum
Pathways_in_cancer
Pancreatic_cancer
Osteoclast_differentiation
Non−small_cell_lung_cancer
Non−alcoholic_fatty_liver_disease_NAFLD
Metabolic_pathways
Melanoma
MAPK_signaling_pathway
Hippo_signaling_pathway
Herpes_simplex_infection
Hepatitis_C
Epstein−Barr_virus_infection
Endocytosis
Central_carbon_metabolism_in_cancer
Biosynthesis_of_antibiotics
Adipocytokine_signaling_pathway
FoldChange
Observed genes
Significance Test
collagen trimer
renal water transport
actin cytoskeleton reorganization
negative regulation of substrate adhesion−dependen ..
cytoskeletal anchoring at nuclear membrane
sterol metabolic process
lipid metabolic process
positive regulation of stress−activated MAPK casca ..
Sphingolipid_signaling_pathway
Proximal_tubule_bicarbonate_reclamation
PPAR_signaling_pathway
Fatty_acid_biosynthesis
B
Active SLE vs HC
obsolete signal transducer activity
protease binding
folic acid binding
apical plasma membrane
nuclear body
nucleolus
integral component of membrane
regulation of protein binding
pyrimidine nucleoside biosynthetic process
O−glycan processing
positive regulation of transcription by RNA polymo ..
transmembrane transport
blood coagulation
cGMP−PKG_signaling_pathway
Viral_carcinogenesis
Tuberculosis
Steroid_biosynthesis
RNA_transport
Pyrimidine_metabolism
Pantothenate_and_CoA_biosynthesis
Nicotinate_and_nicotinamide_metabolism
Mucin_type_O−Glycan_biosynthesis
Glycosaminoglycan_biosynthesis_−_chondroitin_sulfate_dermata ..
Carbohydrate_digestion_and_absorption
Calcium_signaling_pathway
Apoptosis
Alcoholism
nitric oxide binding
UDP−activated nucleotide receptor activity
RNA polymerase III transcription factor binding
signaling receptor binding
phagophore assembly site
activation of cysteine−type endopeptidase activity ..
secondary heart field specification
cellular response to lipopolysaccharide
nucleobase−containing small molecule metabolic pro ..
Olfactory_transduction
Bacterial_invasion_of_epithelial_cells
Amoebiasis
Adrenergic_signaling_in_cardiomyocytes

Supplementary Figure 2 Functional enrichment analysis of related genes in different 5hmC-modified regions. (A) GO analysis of DhMRs enriched in promoter and differentially enriched region-related genes in stable SLE vs. HC. KEGG pathway analysis of DhMRs enriched in promoter and differentially enriched region-related genes. (B) GO and KEGG pathway analyses of DhMRs enriched in promoter and differentially enriched region-related genes in active SLE vs. HC.

Validation SLE vs HC

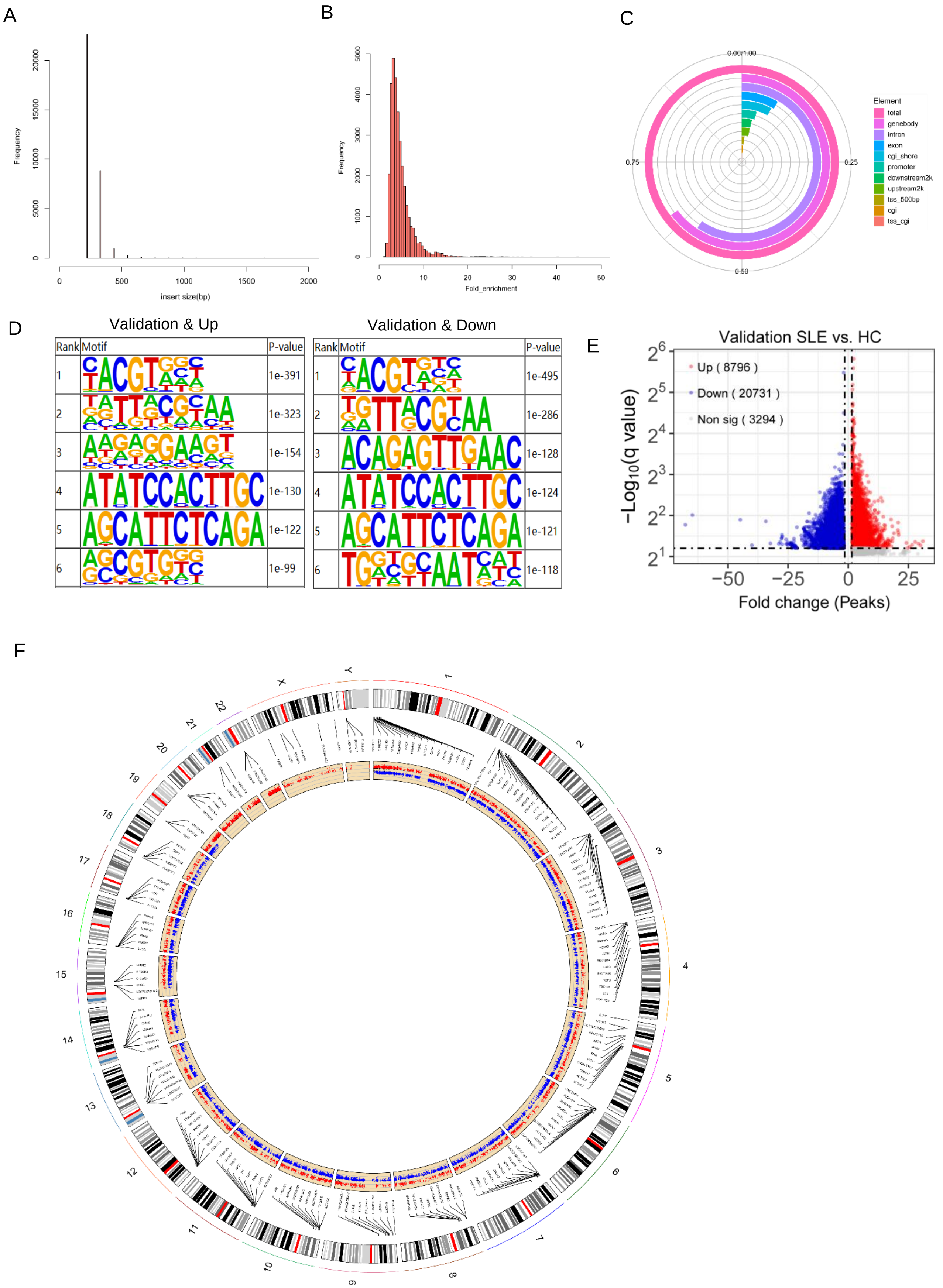

Supplementary Figure 3 Overall genomic distribution and features of DhMRs in validation SLE vs. HC. (A and B) Average frequency of insert size (A) and fold enrichment (B) of DhMRs in validation SLE vs. HC. (C) Average distribution of DhMR ratios across different genomic regions of validation SLE vs. HC. (D) Top six enrichment target motif sequences in DhMRs (including hyper and hypo 5hmC peaks) in validation SLE vs. HC. (E) Volcano plot showing significantly altered 5hmC peaks in validation SLE vs. HC. (F) Distribution across different chromosomes of hyper and hypo DhMRs in validation SLE vs. HC.

Validation SLE vs HC

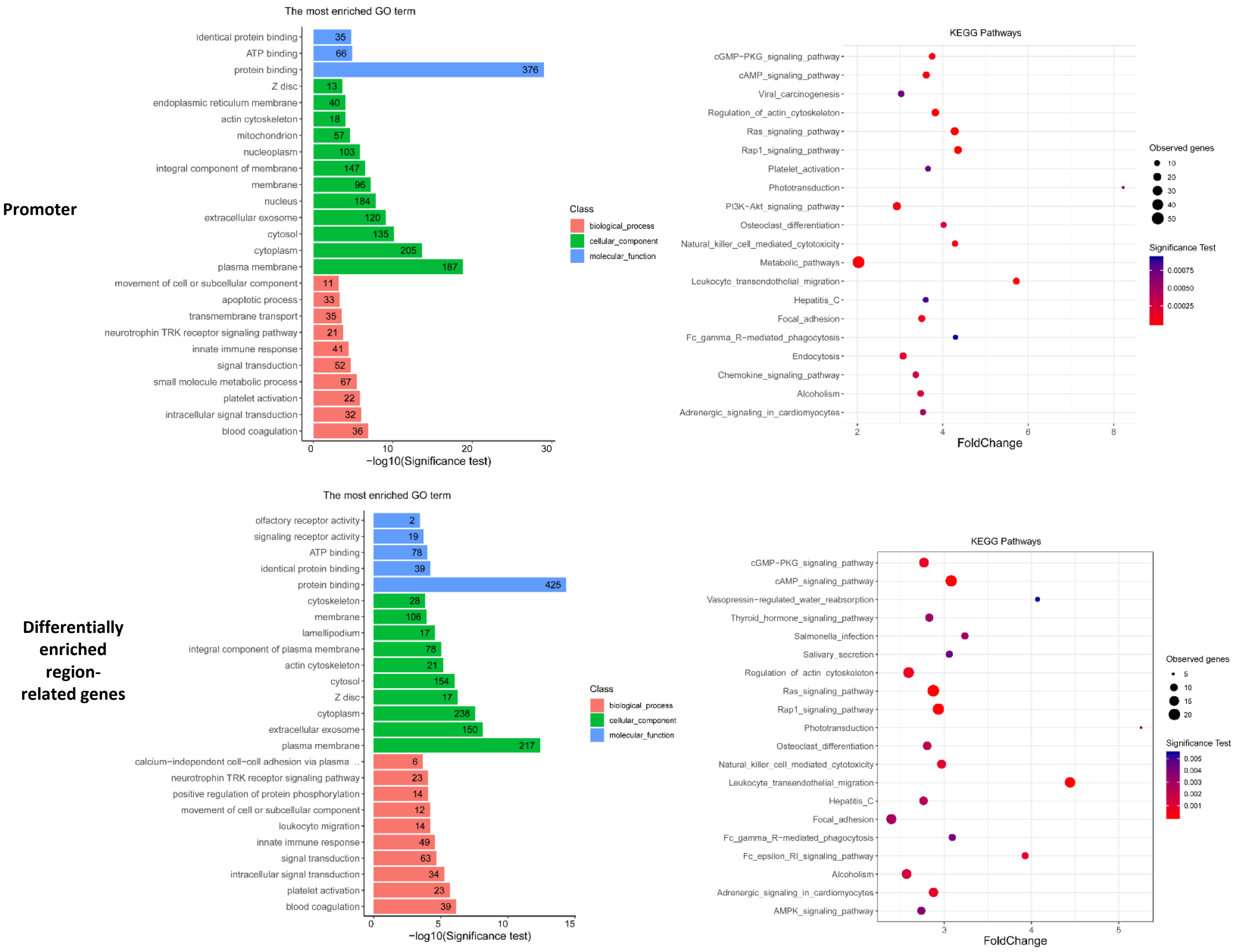

Supplementary Figure 4 GO and KEGG pathway analyses of DhMRs enriched in promoter or differentially enriched region-related genes in validation SLE vs. HC.

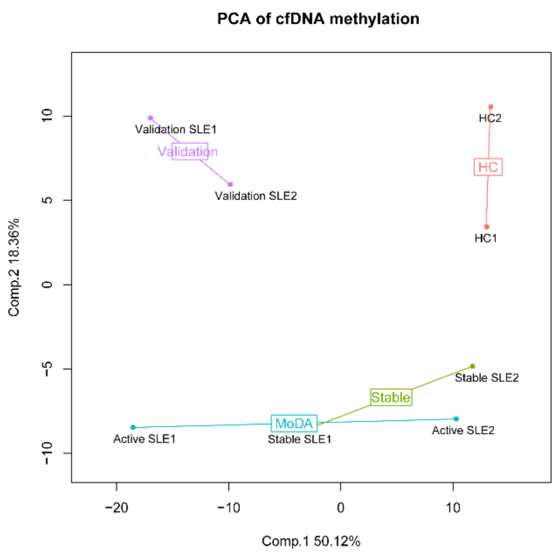

Supplementary Figure 5 Principal component analysis (PCA) showing that cfDNA 5hmC profiles significantly distinguish SLE patients from HCs.

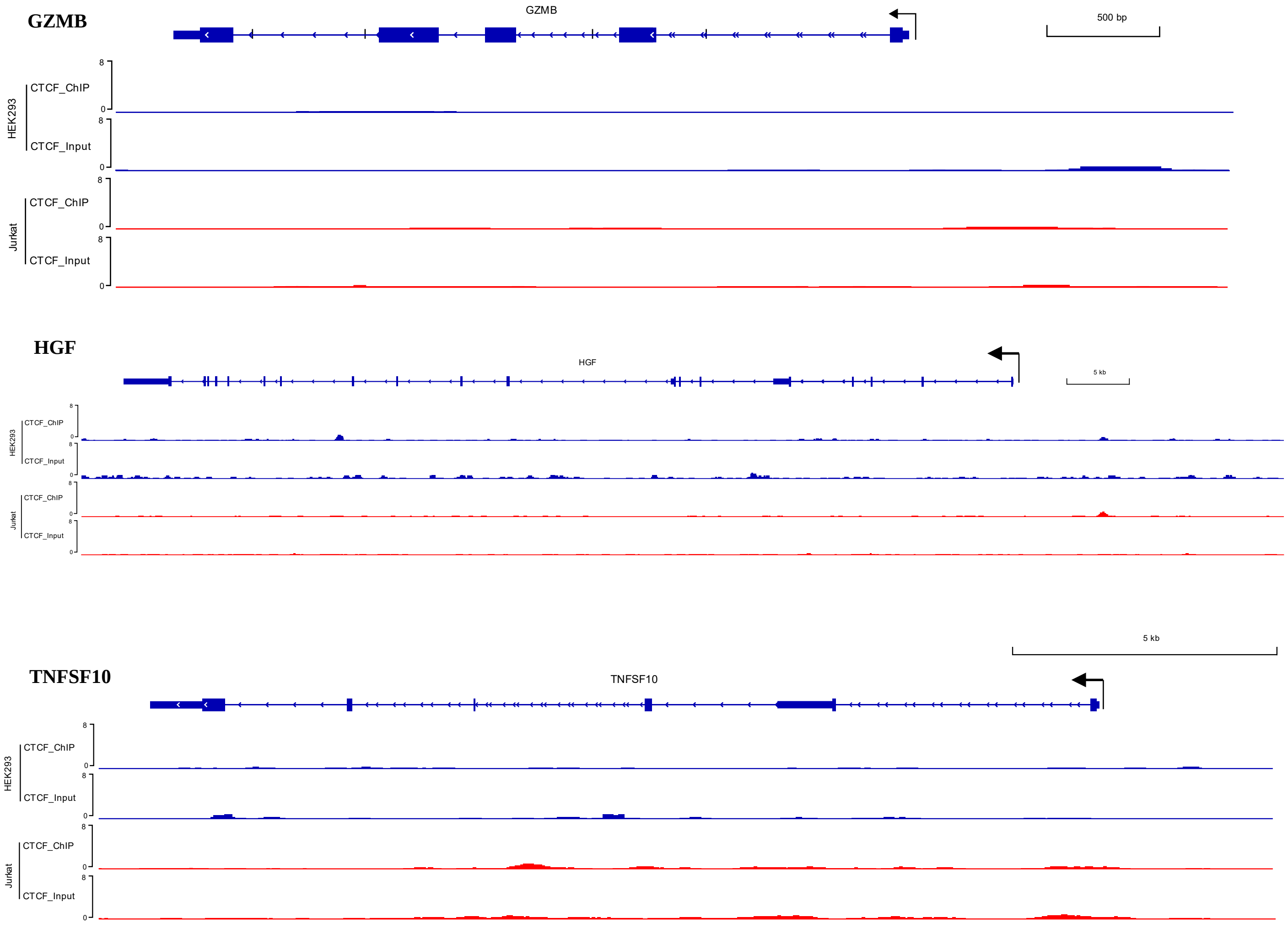

Supplementary Figure 6. Genomic location of CTCF binding regions in the HEK293 or Jurkat cell lines. No CTCF binding regions were observed in GZMB, HGF, or TNFSF10 in the HEK293 or Jurkat cell lines.

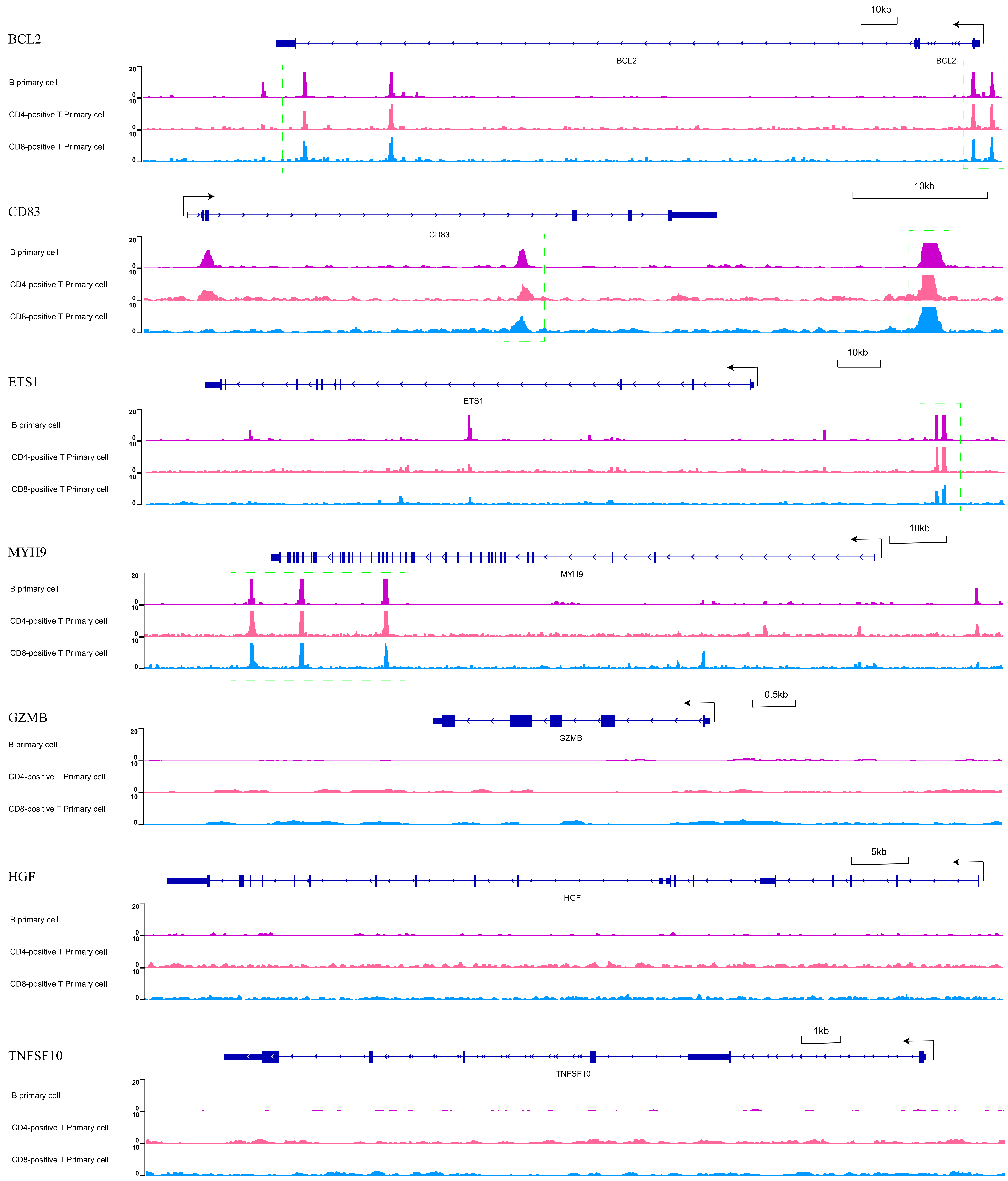

BCL2
10kb
BCL2
BCL2
B primary cell
CD4-positive T Primary cell
CD8-positive T Primary cell
CD83
10kb
CD83
ETS1
10kb
ETS1
MYH9
10kb
MYH9
GZMB
0.5kb
GZMB
HGF
5kb
HGF
TNFSF10
1kb
TNFSF10

Supplementary Figure 7 Genomic location of CTCF binding regions in he primary lymphocytes. Binding regions of the transcription factor CTCF in BCL2, CD83, ETS1, and MYH9 of primary lymphocytes. No CTCF binding regions were observed in GZMB, HGF, or TNFSF10 in the primary lymphocytes.

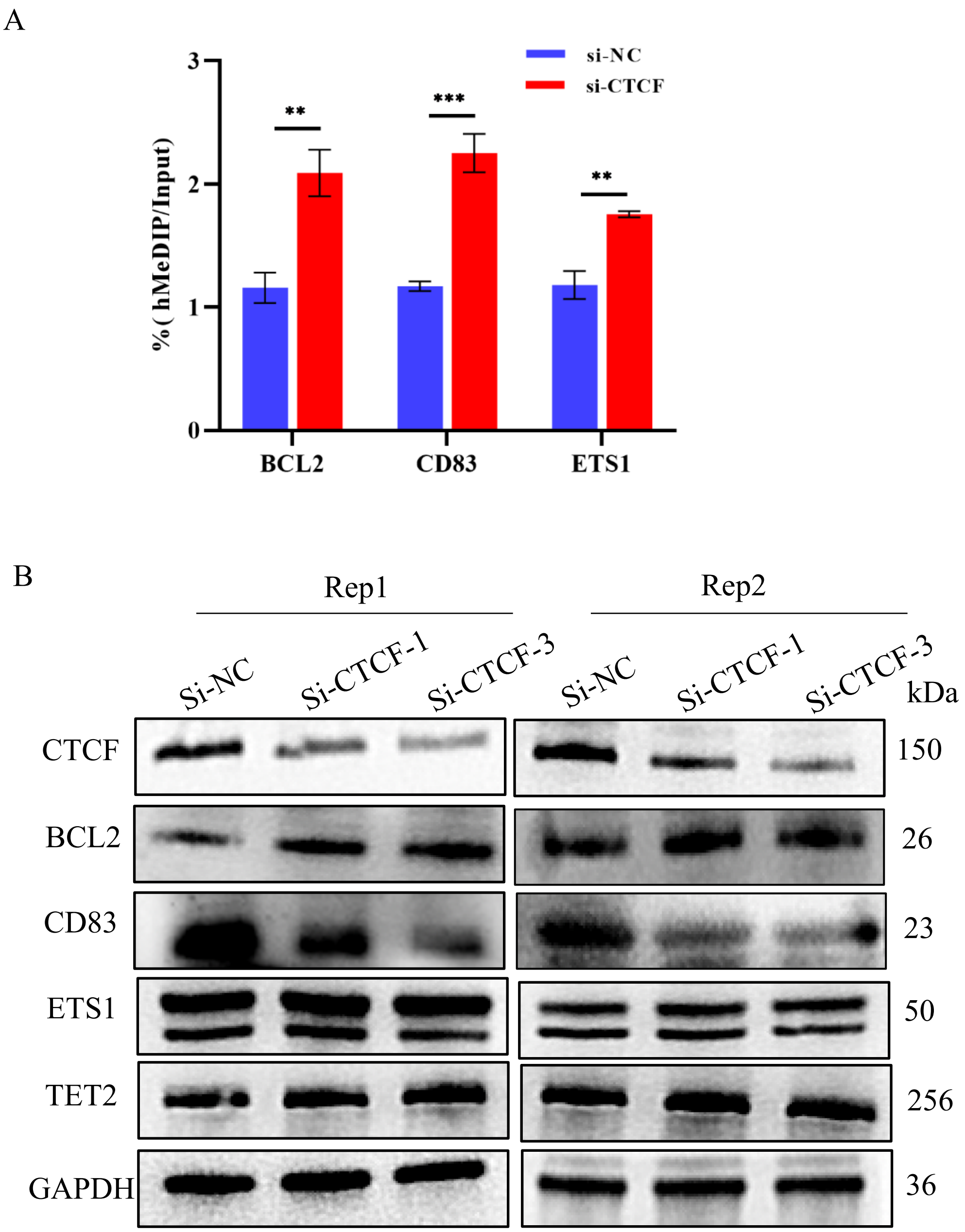

Supplementary Figure 8. CTCF mediates DNA hydroxymethylation and protein expression of potential biomarkers. (A) 5hmC modification levels of BCL2, CD83, and ETS1 (hMeDIP-qPCR) in 293T cells upon CTCF knockdown. (B) Protein levels of candidate DhMG biomarker or TET2 after CTCF knockdown. GAPDH was used as an internal control (**, $P < 0.01$; ***, $P < 0.001$).

Supplementary Tables

Supplementary Table 1. Basic demographic information and SLE Disease Activity Index 2000 (SLEDAI-2K) scores for patients with systemic lupus erythematosus (SLE) included in this study (Mean±SD).

Group	Number(Patient)	Sex (Female)	Age (years)	SLEDAI-2K
SG1	7	5	43.71±20.47	3.43±2.76
SG2	7	7	49.14±11.57	3.00±2.38
AG1	3	3	37.33±5.77	10.00±2.00
AG2	7	7	37.14±9.41	9.86±1.68
VG1	7	7	32.57±7.74	19.14±2.55
VG2	4	4	35.50±5.20	19.00±2.16
HC1	16	16	29.81±3.89	/
HC2	16	16	31.69±8.02	/

Supplementary Table 2. Statistics on sequencing reads data output and quality, as well as alignment statistics with the reference genome in SLE subgroups.

Subgroups	Raw reads	Clean data (reads)	Q20 (%)	Q30 (%)	GC (%)	Mapped rate	Total peaks
SG1	22152948	21524931	97.58	92.69	45.96	99.83	122802
SG2	21756128	21128856	97.56	92.66	46.45	99.84	137964
AG1	19912769	19444636	97.63	92.72	45.43	99.86	115936
AG2	19638906	19035670	97.51	92.53	46.13	99.85	138815
VG1	21076021	20485390	97.56	92.67	45.72	99.83	99026
VG2	20751742	20146146	97.57	92.68	46.63	99.83	111328
HC1	23992197	23248331	97.54	92.62	46.41	99.75	138853
HC2	21229706	20590267	97.56	92.66	46.84	99.84	128857

Supplementary Table 3. Primers for hydroxymethylated DNA immunoprecipitation-quantitative polymerase chain reaction (hMeDIP-qPCR) and quantitative real-time polymerase chain reaction (qRT-PCR).

Experiment	gene	forward primer(5'- 3')	Reverse primer(5'- 3')
hMeDIP-qPCR	BCL2	GCAAGGATAAAGGAAGTACCAA	TTTAGTAGAGATGGGGTTTTGC
hMeDIP-qPCR	CD83	TGAACTCTTGATGAACAGGGG	CGTCCTTGAAGAACGATTGAT
hMeDIP-qPCR	ETS1	CCAGGAGATGGGGAAAGAGG	CAGGTCACACACAAAGCGGTA
hMeDIP-qPCR	MYH9	TTCAAGCGATTCTCCTGCCT	GGGTGGATCACCTGAGGTCA
hMeDIP-qPCR	Positive Control	AGAAGCTCGACCGTCTTGGC	AAACCGAACCGCTACACCCT
qRT-PCR	CTCF	CAGTGGAGAATTGGTTCGGCA	CTGGCGTAATCGCACATGGA
qRT-PCR	MYH9	CAGCAAGCTGCCGATAAGTAT	CTTGTCGGAAGGCACCCAT
qRT-PCR	GAPDH	GTCTCCTCTGACTTCAACAGCG	ACCACCCTGTTGCTGTAGCCAA

Supplemental_Material-online only. Processed data from the enrichment step (MACS peaks).
